# How SARS-CoV-2 Pandemic Changed Traumatology and Hospital Setting: An Analysis of 498 Fractured Patients

**DOI:** 10.3390/jcm10122585

**Published:** 2021-06-11

**Authors:** Marco Brayda-Bruno, Riccardo Giorgino, Enrico Gallazzi, Ilaria Morelli, Francesca Manfroni, Matteo Briguglio, Riccardo Accetta, Laura Mangiavini, Giuseppe Maria Peretti

**Affiliations:** 1 IRCCS Orthopedic Institute Galeazzi, 20144 Milan, Italy; marco.brayda@spinecaregroup.it (M.B.-B.); francesca.manfroni89@gmail.com (F.M.); matteo.briguglio@grupposandonato.it (M.B.); riccacc@gmail.com (R.A.); laura.mangiavini@unimi.it (L.M.); giuseppe.peretti@unimi.it (G.M.P.); 2 Residency Program in Orthopedics and Traumatology, University of Milan, 20122 Milan, Italy; 3 Ortopedia e Traumatologia 3, ASST Centro Specialistico Ortopedico Traumatologico G. Pini–CTO, 20122 Milan, Italy; enrico.gallazzi@gmail.com; 4 U.O.C. Ortopedia e Traumatologia Nuovo Ospedale di Legnano ASST Ovest Milanese, 20025 Legnano, Italy; ilaria.morelli90@gmail.com; 5 Department of Biomedical Sciences for Health, University of Milan, 20122 Milan, Italy

**Keywords:** SARS-CoV-2, fractures, traumatology, hospital setting, health care management

## Abstract

Background: SARS-CoV-2 pandemic is one of the biggest challenges for many health systems in the world, making lots of them overwhelmed by the enormous pressure to manage patients. We reported our Institutional Experience, with specific aims to describe the distribution and type of treated injuries, and the organizational setup of our hospital. Methods: Data of fractured patients admitted for surgical treatment in the time frames 9 March 2020–4 May 2020 and 1 March 2019–31 May 2019 were collected and compared. Furthermore, surgery duration and some parameters of effectiveness in health management were compared. Results: A total of 498 patients were included. Mean age significantly lower age in 2019 and femoral fractures were significantly more frequent 2020. Mean surgery time was significantly longer in 2020. Mortality rate difference between the two years was found to be statistically significant. Time interval between diagnosis and surgery and between diagnosis and discharge/decease was significantly lower in 2020. In 2020, no patient admitted with a negative swab turned positive in any of the following tests for SARS-CoV-2. Conclusions: The COVID-19 pandemic has modified the epidemiology of hospitalized patients for traumatic reasons, leading to an increased admission of older patients with femoral fractures. Nevertheless, our institutional experience showed that an efficient change in the hospital organization, with an improvement of several parameters of effectiveness in health management, led to a null infection rate between patients.

## 1. Introduction

The Novel Coronavirus 2019 (SARS-CoV-2) pandemic is one of the biggest challenges for the National Health Systems (NHS) in modern times. Since the first reported case in December 2019 in Wuhan, Hubei province, China [1], the infection has spread worldwide, with cases reported in all countries [2]. Due to the severity of the clinical picture, during the first pandemic wave more than 20% of patients eventually require admission to Intensive Care Units (ICU) with a long stay [3]. Thus, many of the health systems in the world were overwhelmed by the enormous pressure to manage those patients. Italy was one of the first countries hit by the pandemic first wave, with more than 233,000 total cases, 157,000 healed, 33,000 deaths and 86,000 hospital admissions up to 31 May 2020 (data from the Italian Ministry of Health website: https://www.salute.gov.it/imgs/C_17_notizie_4839_0_file.pdf (accessed on 31 May 2020)) [4].

This unprecedented situation called for unprecedented measures. Starting from 8 March and until 4 May 2020, a ‘Phase 1’ response was implemented: our Region, Lombardy, Italy, was quarantined, with only essential works allowed; gatherings were forbidden, and leaving the house for groceries was allowed once a week only for one member per family [5,6]. Furthermore, this occurrence called for a prompt response also by the Health Ministry and NHS Authorities, pressured to reorganize and rationalize the NHS in order to provide as many ICU beds as possible to treat positive severe ill patients [7]. At the same time, great attention was paid to maintaining the capability to treat other medical and surgical emergencies and limiting as much as possible the risk of intrahospital spreading of the infection. Thus, our Regional Health System was reorganized following the Hub-Spoke model [8,9]. In this context, our Institution was identified as Hub for ‘Minor’ trauma, defined as low-energy, single-district trauma requiring an orthopedic surgical treatment. Hub hospitals were obliged to organize separate pathways, with ‘clean’ areas for non-SARS-CoV-2 patients, and ‘dirty’ areas for SARS-CoV-2 positive patients after hospital triage, in order to treat both type of patients with minimal contagion risk [10]. Furthermore, safety of Healthcare Professionals was a priority, with implementation of a training program to learn how to properly dress and undress with personal protective equipment (PPE: gloves, surgical face masks, goggles, face shield and gowns, as well as items for specific procedures) and to make correct use of them.

In this context, we reported our Institutional Experience during the first wave response to the COVID-19 pandemic, with specific aims to describe the distribution and type of treated injuries, and the organizational setup of our hospital. In particular, we tested these specific hypotheses: (1) the lockdown modified the population behavior, thus modifying the epidemiology and injury distribution of minor trauma patients; (2) our activity produced a high level of quality and effectiveness through the evaluation of technical times in the treatment of trauma patients; (3) the internal organizational system was effective in limiting in-hospital spread of the disease among patients and healthcare workers.

## 2. Materials and Methods

### 2.1. Study Population and Methods

The electronic registry of our institution was searched for patients with fractures admitted for surgical treatment in the time frames 9 March 2020–4 May 2020 (pandemic group) and 1 March 2019–31 May 2019 (non-pandemic group). Patients with nasopharyngeal swab positive for SARS-CoV2 were included as well. The anonymized demographic and clinical data of all included patients were extracted. Age, fracture distribution, differences in death rate and discharge type were compared between the two groups. Furthermore, surgery duration and the time intervals between diagnosis and surgery and between diagnosis and discharge/death during hospital stay were analyzed as parameters of clinical efficiency and compared.

### 2.2. Hospital Organization

Our facility is located in a 9-story building, with a cave-square shape. The two basement floors host: Emergency and Radiology departments, Clinical Chemistry and Microbiology Laboratories, offices, Nuclear Magnetic Resonance Unit, locker rooms, storages and research laboratories. Both basement floors are connected to the internal yard. Management offices, a cafeteria, several offices for outpatient visits and a wide hall with front desks are present on the ground floor. First floor hosts outpatient rooms, the dentistry unit, the rehabilitation gym and some operatory rooms for day-hospital surgery. The other floors are entirely dedicated to surgical and rehabilitation inpatients wards, usually hospitalized in double rooms. Some areas on the 5th and 6th floors include operatory rooms. The Intensive Care Unit (ICU) is also on the 5th floor, while the remaining part of the 6th hosts some offices and research labs.

With the beginning of COVID-19 pandemic in March 2020, the following changes have been implemented to limit in-hospital disease spread:

- Only non-deferrable outpatient visits and radiological exams were allowed, and the access of patients’ relatives was possible only in case of real need, to reduce hospital overcrowding. Social distancing rules have been applied in all the waiting rooms, spacing and reducing seats with dedicated elevators.
- The second floor was entirely converted into a COVID-19 unit, hosting both hospitalized patients with SARS-CoV-2-related respiratory disease and positive patients with surgical fractures (during the acute surgical care). The 3rd floor was transformed into a pre-COVID unit, hosting surgical patients admitted trough emergency unit and before the results of nasopharyngeal swab. Based on the results, they were sorted into the COVID unit of the 2nd floor or into the clean area. The passage of healthcare workers (HCWs) was allowed only from the pre-COVID to the COVID unit, never inversely, according to a gradient from suspect to ascertained cases. As the number of positive cases decreased, the pre-COVID unit was transferred into a small, separated area on the 2nd floor.
- The 4th floor was the ‘COVID-free’ area, entirely dedicated to surgical inpatients with negative swabs.
- The operatory rooms on the 5th story were converted into COVID-ICU.
- The 6th floor hosted the COVID-free ICU, while the surgical unit was split into non-communicant COVID and COVID-free surgical units, with separate accesses.
- All the COVID areas were provided with changing rooms with showers for HCWs.
- All the ward rooms, generally hosting two patients, were transformed to host only one patient.
- Patients transfers between the COVID areas (COVID unit, operatory room and ICU) and between the COVID-free areas occurred through different pathways, corridors and elevators. Corpses were transported through the COVID pathway.
- No courtesy visits on assistance to hospitalized patients were allowed anymore, except for pediatric patients with one parent swabbed and not allowed to leave the room.
- HCWs access was guaranteed only through the main entrance. Outpatients entered the hospital through the main entrance; when needing hospitalization, patients entered through the emergency department (ED). Due to the high prevalence of SARS-CoV-2 in Lombardy, all patients arriving at hospital filled in a short anamnestic questionnaire regarding respiratory symptoms or possible personal contacts with cases of COVID-19. Fever screening with thermal cameras was mandatory for all people entering the hospital: if allowed (with temperature <37.5 °C), they were provided with a new surgical mask, that both workers and patients had to wear inside the hospital (even during oxygen therapy or in the operatory rooms, if tolerated).

### 2.3. SARS-CoV-2 Testing

In our facility, each patient underwent a SARS-CoV-2 nasopharyngeal swab at admission (even when transferred from other hospitals), after 3 days from admission and then every 5 days until discharge. The rate of COVID-19-negative patients at admission, whose SARS-CoV-2 nasopharyngeal swabs turned positive during hospitalization, was analyzed. This was attributed to possible in-hospital disease spreading only if the swabs turned positive 3 days from the negative test at admission.

### 2.4. Statistical Analysis

Statistical analyses were performed using GraphPad Prism (version 8, GraphPad Prism Software Inc.). Contingency tables with Chi-square test calculation were used to compare categorical variable distribution between the two groups. For 2 by 2 contingency tables, odds ratios (OR) were calculated, and reliability expressed through 95% confidence intervals (CI). Unpaired Student’s *t*-test was used to compare continuous variables, and results reported as t(degrees of freedom), *p*-value. Statistical significance was set at *p* ≤ 0.05. The raw data used to support the findings of this study are included within the Supplementary Information File as a Microsoft Excel worksheet.

## 3. Results

**Figure 1:**
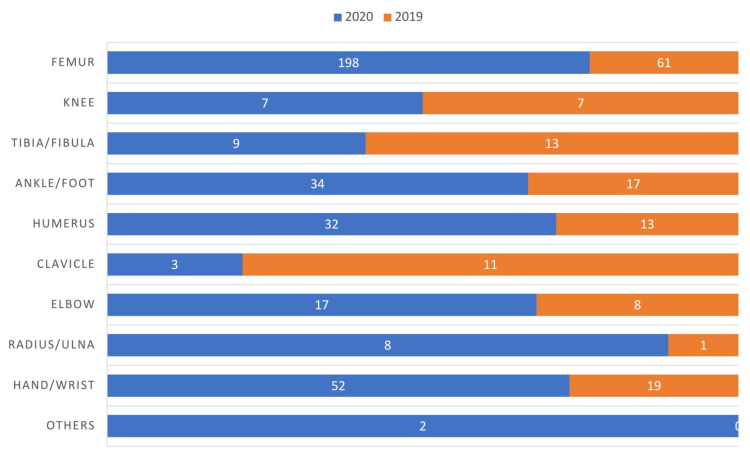
Typification of fractures. Femoral fractures were significantly more frequent in 2020 (*p*-value = 0.0137).

A total of 498 patients were included, 146 patients in 2019 and 352 in 2020, overall reporting 512 fractures (153 in 2019 and 359 in 2020). Indeed, 14 patients reported more than one fracture. The overall mean age was 67 ± 23 years, with a significantly lower age in 2019 (61 ± 24.5 years versus 69 ± 21.5, t(496) = 3.6251, *p*-value = 0.0003). Fracture distribution is reported in Figure 1.

Femoral fractures were significantly more frequent in the pandemic group (181 out of 352 in 2020 versus 57 out of 146 fractures in 2019, *p*-value = 0.0137). Interestingly, mean surgery time was significantly longer in 2020 rather than in 2019 (89 ± 36.7 min and 78 ± 31.3 min, respectively; t(496) = 3.1739, *p*-value = 0.0016). After surgery, 89 patients (61%) from the non-pandemic group were discharged home, 56 (38,4%) were transferred into rehabilitation facilities and one (0,7%) into nursing home. Similarly, 212 (60,2%) patients from the pandemic group were discharged home, 124 (35,2%) transferred into rehabilitation facilities and 6 into nursing homes (1,7%), while 10 patients died during their hospital stay (2,8%). The mortality rate difference between the two years was found to be statistically significant (*p*-value = 0.0389) (Table 1). Among the 10 deaths, 3 patients died from serious comorbidities and 7 from COVID-related thromboembolic events. More precisely, 4 patients died between day 0 and day 2 and 6 patients between day 8 and day 10.

**Figure 2:**
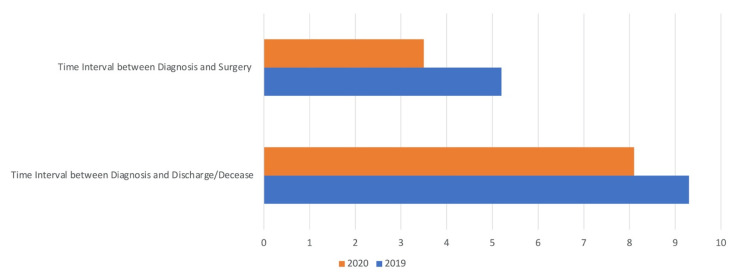
Parameters of effectiveness in health management. Time interval between diagnosis and surgery and between diagnosis and discharge/decease were significantly lower in 2020 (respectively, *p*-value = 0.0007 and *p*-value = 0.0284).

Furthermore, the time interval between diagnosis and surgery in 2020 was 3.5 ± 4.2 days, in contrast to 2019 when it was 5.2 ± 6.7 days (t(496) = 3.4128, *p*-value = 0.0007). Additionally, the time interval between diagnosis and discharge/decease was significantly lower in 2020 compared to 2019 (8.1 ± 5.2 days and 9.3 ± 6.3 days, respectively; t(496) = 2.1988, *p*-value = 0.0284) (Figure 2). In the pandemic group, no patient admitted with a negative swab turned positive in any of the following tests for SARS-CoV-2.

## 4. Discussion

Our findings revealed that, with the structural and organizational changes adopted in our hospital, our facility faced, with success, the first pandemic wave increase in hospital admissions due to trauma Hub designation. During the first pandemic wave, an epidemiological change, compared to 2019, was found with regards to the admitted patients, presenting a higher mean age and mainly femoral fractures. Despite the patients admitted during 2020 were mainly frail older adults, no in-hospital COVID-19 spreading was found. This could be explained with the rigid separation between COVID and COVID-free areas at our facility, close monitoring with repeated nasopharyngeal swabs during hospitalization and reduced time intervals from diagnosis to surgical treatment and to discharge. Furthermore, the increase in surgical time could reflect first of all the strict application of PPE wearing protocols, in order to reduce SARS-CoV-2 diffusion.

The first aim of our study was to analyze the variation of orthopedic treatment request during quarantine: epidemiological data are valuable, both for surgeons and for stakeholders, in order to optimize the personnel and instrumentation required to manage specific types of fractures. We previously reported [10] that during the March 2020 lockdown the percentage of ER admissions as white and green code (walking wounded) markedly decreased when compared to the same time span during 2019. Similar reduction was reported in several countries in the world during the SARS epidemic [11,12]. On the contrary, admission triages as yellow and red code (urgent patients, including femoral fractures) markedly increased in same time frame. This higher volume of fracture-related admission could be a consequence of the centralization of minor trauma to Hub hospitals during the emergency. Furthermore, it could be due to a decreased home-assistance to older adults by the caregivers, in order to avoid disease spreading, during the lockdown. Other studies evaluated the impact of lockdown in trauma admission. Giorgi et al. reported an increase in high energy traumas that caused vertebral fracture at the early stage of the March lockdown compared to the same time span of the previous year in a major trauma hub [13]. Ogliari et al. [14] reported a decrease in overall fractures admitted to a Fracture Clinic, while observing the same amount of hip fractures admitted during lockdown when compared to prior period before the lockdown; the authors evidenced a change in epidemiology of injury due to the reduced traffic and work activities (lockdown), thus suggesting hospitals to prepare accordingly. Conversely, Poggetti et al. [15] reported the same amount of hand and wrist fractures prior and during the lockdown; they, however, noted a change in etiology, with less sport and traffic related injuries and more domestic accidents, with a shift towards patients with older age. Interestingly, Bram et al. [16] reported a 2.5 SD fold decrease in pediatric fractures during the lockdown, mainly due to cessation of organized sport and the prohibition of playground use. In this context, our paper offers a different perspective, due to the Hub organization of the Italian NHS: since most of the fractures were shifted to the Hub hospitals, with only two minor trauma Hub serving a metropolitan area of roughly 1.5 million inhabitants, our data are less biased by the pathology ‘dispersion’ and could better reflect the real-life scenario. The second aim of this study was to evaluate some parameters of hospital clinical efficiency in a different scenario, as the surgical times and the time frames passed from diagnosis to surgery and from diagnosis to discharge. Surgical times were longer in the pandemic group, and this could be explained by the time needed to wear the adequate PPEs, as well as by the fact that PPEs and apprehension due to possible intraoperative HCWs contagion may have slowed down the global surgical times (admission to OR, patient preparation and positioning and post-surgical management). On the other hand, both diagnosis-surgery and diagnosis-discharge times were reduced, and this could be considered as an overall improvement in clinical efficiency during the pandemic. This may be due to the rigid internal organization, as the protocols applied may have automatized and quickened the everyday procedures. Unluckily, since the deceased patients were considered as discharges, the death of 10 patients surely contributed to reduce the diagnosis-discharge time. On the other hand, the higher mortality rate is aligned with other studies reporting an increased mortality rate for COVID-19 fractured patients [17,18,19]. The third aim of this paper was to evaluate the effectiveness of the applied hospital measures to reduce the risk of contagion between patients. Overall, we had no cases of swabs turned positive among patients hospitalized in the ‘clean’ area. This was an excellent result, underlining the effectiveness of the measures adopted. In fact, COVID-19 diffusion among hospitalized patients was one of the most severe issues to manage during the first Italian outbreak [20,21]. During epidemics, hospitals and nursing homes could become real pitfalls for the disaster response phase [20]. Especially at the beginning of a pandemic, hospitals, that are already hosting frail people, such as older adults or patients with several comorbidities, are overcrowded with an increasing number of contagious people. If the viral pathogen is not rapidly recognized, and preventive measures are not immediately implemented, nosocomial transmission is a dangerous consequence both for inpatients and medical personnel [22].

The currently ongoing SARS-CoV-2 pandemic is the biggest challenge that the National health Systems handled in the last century. Most of the European countries faced the early 2020 outbreak of March and April, and Italy was the first and most affected country [23]. Afterwards, an overall case reduction during summer was perceived [24]. Therefore, during the fall, the direst predictions were confirmed, and a new outbreak affected the European Union. In Italy, the total number of cases registered in October was almost 300,000, surpassing by far the total number of cases registered in March and April [4]. Furthermore, with an increasing number of patients currently requiring ICU, the NHS was again on the verge of collapse [25]. Orthopedic practice was also markedly affected by the pandemic. Elective surgical activity is currently limited only to cases with severe pain and functional limitation, or risk of disease progression. As described in a previous paper [13], the NHS is again being reorganized to face the ongoing second emergency, with the goal of rationalizing care and reducing the risk of in-hospital contagion. Indeed, most of trauma occurring in our metropolitan area are shifted to three ‘Hub’ hospitals, one for major traumas and two for minor traumas. Similar organizational models are currently being implemented in other parts of the country and across Europe [26]. In this context, it is of utmost importance to report the experience acquired during the first outbreak. In addition to the encouraging results regarding the management of a health emergency, our results appear valuable in the possible future comparison with what happens during the further COVID-19 waves.

## 5. Conclusions

This work reports our institutional experience during the first wave of SARS-CoV-2 pandemic when we were able to set an optimal organization of the hospital, an example to consider and eventually adapt in the light of specific needs. The new hospital assessment included an increase of dedicated beds, ORs and health-care personnel, thus leading to an improvement of numerous parameters of effectiveness in health management. Furthermore, we showed an ideal inpatient protection, with a rigid separation between COVID and COVID-free area, a strict personnel flow from clean to COVID areas and close inpatients monitoring with serial swabs, which led to a null infection rate between patients. These organizational changes are also easily reproducible in other little single-building multistory facilities, and should be considered in order to limit in-hospital disease spreading.

## Figures and Tables

**Table 1:** Differences in patients admitted between 2019 and 2020.

	2019	2020	*p*-Value
Mean age (years)	61 ± 24.5	69 ± 21.5	0.0003
Femoral fractures/total fractures	57/146	181/352	0.0137
Mean surgical time (minutes)	78 ± 31.3	89 ± 36.7	0.0016
Diagnosis-to-surgery time (days)	5.2 ± 6.7	3.5 ± 4.2	0.0007
Diagnosis-to-discharge/decease time (days)	9.3 ± 6.3	8.1 ± 5.2	0.0284
Deceased patients	0/146	10/352	0.0389

## Data Availability

The data presented in this study are available on request from the corresponding author. The data are not publicly available due to privacy.

## Supplementary Materials

The following are available online at https://www.mdpi.com/article/10.3390/jcm10122585/s1.
